# Social Assistance Programs and Birth Outcomes: A Systematic Review and Assessment of Nutrition and Health Pathways

**DOI:** 10.1093/jn/nxab292

**Published:** 2021-09-29

**Authors:** Jef L Leroy, Bastien Koch, Shalini Roy, Daniel Gilligan, Marie Ruel

**Affiliations:** Poverty, Health, and Nutrition Division, International Food Policy Research Institute, Washington, DC, USA; Poverty, Health, and Nutrition Division, International Food Policy Research Institute, Washington, DC, USA; Poverty, Health, and Nutrition Division, International Food Policy Research Institute, Washington, DC, USA; Poverty, Health, and Nutrition Division, International Food Policy Research Institute, Washington, DC, USA; Poverty, Health, and Nutrition Division, International Food Policy Research Institute, Washington, DC, USA

**Keywords:** social assistance, birth outcomes, lower birth weight, small-for-gestational age, pregnancy, systematic review

## Abstract

**Background:**

Poor birth outcomes are an important global public health problem. Social assistance programs that provide cash or in-kind transfers, such as food or vouchers, hold potential to improve birth outcomes but the evidence on their effectiveness has not been reviewed.

**Objectives:**

We systematically reviewed studies that used experimental or quasi-experimental methods to evaluate the impacts of social assistance programs on outcomes in low- and middle-income countries.

**Methods:**

The Grading of Recommendations, Assessment, Development and Evaluations (GRADE) system was used to assess the certainty of the evidence for birth weight and neonatal mortality (most common outcomes reported). We summarized the evidence on hypothesized nutrition and health pathways of impact.

**Results:**

We included 6 evaluations of 4 different cash transfer programs and 1 evaluation of a community-based participatory learning and action program that provided food and cash transfers. The 4 studies that assessed birth weight impacts found significant (*P* < 0.05) effects ranging from 31 to 578 g. Out of 3 studies that assessed neonatal mortality impacts, 2 found significant effects ranging from 0.6 to 3.1 deaths/1000 live births. The certainty of the evidence for both outcomes was rated as very low due to several methodological limitations. In terms of potential pathways, some studies documented positive effects on maternal diet, antenatal care (ANC) utilization, and delivery in a health facility.

**Conclusions:**

Better-designed evaluations are needed to strengthen the evidence base on these programs. Evaluation studies should elucidate underlying mechanisms of impact by including outcomes related to maternal diet, ANC seeking, use of skilled delivery, and women's empowerment in nutrition and health domains. Studies should also assess potential unintended negative consequences of social assistance, such as reduced birth spacing and excess pregnancy weight gain.

See corresponding editorial on page 3599.

## Introduction

Poor birth outcomes remain an important global public health problem. In 2012, an estimated 23.3 million infants or 19.3% of live births were born small-for-gestational-age in low- and middle-income countries (1). More than 20 million children (or 14.6% of live births) were born with low birth weight (birth weight below 2500 g) in 2015 (2). Poor birth outcomes are strongly associated with child wasting and stunting (3), increase the risk of dying during the neonatal period and later in childhood, are associated with neurocognitive impairment, and are believed to increase risks of noncommunicable diseases, including cardiovascular disease and insulin resistance or type 2 diabetes, later in life (4). The global low birth weight prevalence is steadily decreasing, but progress has been too slow to meet the World Health Assembly target of reducing the number of live births with a low birth weight by 30% between 2012 and 2025 (2). The cost of inaction is high. It has been estimated that without additional interventions to reduce the prevalence of poor birth outcomes, there will be an additional 49 million neonatal deaths, 52 million stillbirths, and 99 million children who will not reach their cognitive development potential by 2035 (4).

Maternal undernutrition is an important contributor to adverse birth outcomes (1). Both micro- and macronutrients are required for the physiological changes and increased metabolic demands during pregnancy, as well as for fetal growth and development. Inadequate intake of vitamins and minerals is known to negatively affect the health, function, and survival of the mother and fetus. Maternal iodine deficiency is associated with delays in neural, intellectual, and physical development; folate deficiency is associated with a higher risk of neural tube defects; vitamin A deficiency in mothers can lead to night blindness; and iron deficiency anemia is believed to lead to low birth weight and increased perinatal mortality (5).

Proven approaches to improving diets and nutrient intakes during pregnancy include nutrition counseling, iron/folic acid or multiple micronutrient supplements, and balanced energy and protein supplementation (6–9).

In 2016, the WHO issued new recommendations on antenatal care (ANC), which included a set of nutrition interventions focused on improving diets and nutrient intake during pregnancy (10). In food-insecure populations, balanced protein energy supplementation and counseling were recommended as interventions to improve maternal diets. Given the high costs of these supplements and the challenges of reaching all pregnant women, however, the WHO recommended research on the effectiveness of alternative approaches, such as cash transfers and vouchers, to increase energy and protein intakes in food-insecure settings.

The WHO recommendation implies a research agenda aimed at understanding whether social assistance programs, which provide transfers—either cash or in-kind—to poor households, offer a promising platform to leverage improvements in birth outcomes. These programs are becoming increasingly popular in low- and middle-income countries, especially in food-insecure areas. Their scale and targeting to vulnerable households make them promising for reaching nutritionally at-risk populations. Moreover, there are a number of plausible nutrition and health pathways by which social assistance programs could improve birth outcomes. To develop a research agenda to rigorously assess the effectiveness of such approaches in improving birth outcomes, it is useful to first take stock of what existing evidence shows about program impacts and pathways. Thus, the first objective of this paper was to systematically review the evidence on the impacts of social assistance programs, measured by experimental and quasi-experimental methods, on birth outcomes in low- and middle-income countries. The second objective was to explore evidence on nutrition, health, and other pathways of impact.

### Nutrition and health pathways by which social assistance programs may improve birth outcomes

Cash and food transfers can improve household food availability and, consequently, women's diet and nutritional status (11). The programs’ targeting strategies, which often involve giving the transfers directly to women, may empower women and increase their control over resources and their decision-making power related to their own nutrition and health (12–14). Transfers can also improve women's psychosocial health (12). They may reduce financial barriers to seeking ANC and skilled birth attendance. Attending ANC and delivery in a health facility may also be part of the conditions to receive the transfer. Behavior change communication (which is part of some programs) and increased contact with health staff may provide women and their families with information on adequate health and nutrition behaviors, as well as greater social capital (15, 16). Finally, the social assistance program can be used as a platform to distribute other benefits, such as nutrient supplements.

The impact of social assistance programs on birth outcomes could also be negative. Transfer programs that are conditional on being pregnant or that provide per-child benefits may create an incentive for women to become pregnant again (or sooner), though most studies find only small effects or no effects (17–19). Shorter birth spacing is associated with negative birth outcomes (20). The requirement to give birth in specific public health facilities [such as in India's Janani Suraksha Yojana program (21)] may reduce the quality of care women receive if the quality of the private care they would have otherwise received was higher or if the increase in demand for public health care is not met with an adequate increase in supply.

## Methods

This systematic review was conducted following the Preferred Reporting Items for Systematic Reviews and Meta-Analyses (PRISMA) statement (22). The review protocol was not registered.

### Inclusion criteria

The review was limited to empirical studies that used experimental or quasi-experimental designs to evaluate the impact of social assistance programs on birth outcomes in middle- or low-income countries. Social assistance programs were defined broadly and included programs providing cash or in-kind transfers such as food or vouchers. Interventions that only provided dietary supplements or fortified products such as lipid-based nutrient supplements targeted to pregnant women were excluded from the review. Studies were required to assess program impacts on at least 1 of the following outcomes: birth weight, low birth weight (defined as weight <2500 g), being small for gestational age, and neonatal mortality (mortality measured within 1 mo after birth).

The review was limited to articles published in English in or after 2000. Studies had to be original research articles. We thus excluded editorials, commentaries, and similar non–primary research articles. We further excluded studies without a comparison group.

### Data sources and search strategies

Details on the search strategy are provided in the **Supplemental Methods**.

### Study selection and analysis

We first reviewed the titles of all identified articles to exclude studies that were clearly outside the scope of the review. We subsequently read the abstracts to exclude papers not meeting the criteria for study scope and study type. Finally, we read the full text to exclude ineligible papers missed in the first 2 steps. The included papers were summarized in tabulated form using the following categories: country, intervention (including the eligibility criteria), sample characteristics (data sources and years, sample size), evaluation design and analytic method (definition of treatment, outcomes assessed, statistical methods), and results by outcome. Authors of included studies were contacted to provide additional details on their analyses.

Due to the small number of studies identified and the heterogeneity in the evaluation designs used, no quantitative synthesis of findings was conducted.

### Assessment of quality of evidence

Two independent reviewers (JLL and BK) used GRADE (Grading of Recommendations, Assessment, Development and Evaluations) to assess the certainty of the evidence for birth weight and neonatal mortality, the 2 outcomes that were reported by at least 3 studies. As opposed to a study-level assessment, GRADE is used to reflect the extent of confidence in the outcome-level effect estimates, that is, considering the entire body of evidence (23–35). Any discrepancies between reviewers were resolved by discussion. Since both randomized and nonrandomized studies were included, a formal assessment and comparison of study-level bias was not possible. Concerns about key sources of bias, such as attrition, lack of control for confounding, selection of participants, and selective reporting of outcomes, are presented in **Table 1** and discussed in the text.

**TABLE 1:** Characteristics of the studies evaluating the impact of social assistance on birth outcomes^1^

Country, name of program, reference trial registration	Program objectives, eligibility and targeting, pregnancy focus	Program description^2^	Evaluation design, sample characteristics, analysis	Implementation issues^3^, exposure^4^	Descriptive statistics and study impact^4^	Concerns
India *JSY* Lim et al., 2010 (21) Registration: None	**Objectives**: Reduce maternal and neonatal deaths by incentivizing women to give birth in health facilities. **Eligibility criteria and targeting:** All women in high-focus states (those with low in-facility birth coverage); in non–high focus states, only for first 2 live births, and only if woman below government poverty line or from scheduled caste or tribe.Cash disbursed to the mother immediately at the facility itself and within a week of delivery. **Pregnancy focus:** program specifically aimed at improving pregnancy outcomes.	One-time cash transfer to women, conditional on giving birth in public or accredited private health facilities (transfers ranged from ∼USD 13 to USD 31, depending on the state and urban vs. rural). Smaller cash transfer also provided for home deliveries to women below poverty line for first 2 births (USD 11) (continuation of existing government program). Community health workers helped women receive ANC services during pregnancy and received USD 4–13 for each birth attended.	**Design**: Quasi-experimental, using individual matching (births receiving JSY and not receiving JSY), before-and-after, and district-level DID. **Data**: Two rounds of the District-Level HH Survey: DLHS-2 2002/04 (before JSY) & DLHS-3 2007/08 (2 to 3 y after rollout). **Sample:** DLHS used multistage stratified sampling (∼1,000 HHs per district in DLHS-2; ∼1,000–1,500 HHs per district in DLHS-3). Depending on analysis: *1*) most recent birth after 1 Jan 2004 in DLHS-3; *2*) most recent birth in DLHS-2 and -3; and *3*) consistently defined (across both data sets) district aggregates. ***N***: Matching: 158,049; with-vs.-without: 370,995; DID: 580. **Analysis**: *1*) Exact matching of JSY and non-JSY births from DLHS-3 on state, urban/rural, poverty, wealth quintile, caste, education, parity, age, followed by logistic regression on matched data, controlling for HH-level covariates; *2*) with-vs.-without comparison using logistic regression on the pooled DLHS-2 and -3 and controlling for HH-level covariates; and *3*) district-level DID using OLS on both DLHS rounds and controlling for district-level covariates; Tx in the DID analyses defined as the district-specific proportion of all women giving birth who received JSY cash.	**Implementation issues:** Goal of reaching poorest women only partly reached: odds of receiving cash tended to be higher for scheduled castes and tribes and other backward groups, but also for educated women, young women, and women in the 3 middle wealth quintiles. Lower uptake by poorest and least educated women partly explained by hard-to-reach populations, restricted access to health facilities, cultural barriers and financial incentives to deliver at home. Differential uptake by state partly due to variation in program awareness and access to infrastructure. **Exposure:** % of women receiving JSY payment ranged from 7% to 44% at the state level, but participation among those eligible not reported. No data on whether women who reported receipt of cash were, in fact, aware of the program or had been encouraged to deliver in a facility.	**Birth outcomes ^5^** **Perinatal deaths/1000 pregnancies** BL: 42. 0Impact: matching: –3.7 (*P* < 0.05); with-vs.-without: –4.1 (*P* < 0.05); DID: NS **Neonatal deaths/1000 live births** BL: 33.6 Impact: matching: –2.3 (*P* < 0.05); with-vs.-without: –2.4 (*P* < 0.05); DID: NS (effect largest in non–high focus states) **Other outcomes** ^6^ **Three ANC visits** BL: 45.7% Impact: matching: +10.7 pp (*P* < 0.05); with-vs.-without: +11.1 pp (*P* < 0.05); DID: +10.9 pp (*P* < 0.05) **In-facility births** BL: 41.0% Impact: matching: +43.5 pp (*P* < 0.05); with-vs.-without: +43.9 pp (*P* < 0.05); DID: +49.2 pp (*P* < 0.05) **Skilled birth attendance** BL: 48.7% Impact: matching: +36.6 pp (*P* < 0.05); with-vs.-without: +36.2 pp (*P* < 0.05); DID: +39.3 pp (*P* < 0.05)	Unobserved confounding due to nonexperimental nature of study cannot be ruled out. Reports show that some women may have been encouraged to use JSY (and thus incentivized to deliver in a health facility) but did not receive the transfer after in-facility delivery. They were, hence, incorrectly classified as comparison group (potential downward bias of treatment effect). Reverse causality cannot be excluded as women received cash because of an in-facility birth (and did not deliver there because of the cash), i.e., they were not aware of the incentive. It cannot be ruled out that the accuracy of the mortality reporting was systematic, i.e., related to program uptake. Potential underestimation of program effects due to financial incentive for home deliveries (more women might have delivered in an accredited facility in the absence of this incentive).
India *JSY* Powell-Jackson et al., 2015 (40) Registration: None	**Objectives:** See above [Lim et al. 2010 (21)] **Eligibility criteria and targeting:** *ibidem*. **Pregnancy focus:** *ibidem*.	See above [Lim et al. 2010 (21)]	**Design**: Quasi-experimental design; district-level DID. **Data**: Two rounds of the household-level DLHS-2 2002/04 (before JSY rollout) & DLHS-3 2007/08 (2 to 3 y after rollout). **Sample:** Live births and deliveries among currently married women in the ∼4 y preceding the survey in 587 districts. ***N***: 507,622 currently married women in DLHS-2 and 643,944 married women in DLHS-3 contributing 429,443 live births (mortality outcomes); 342,875 deliveries (utilization outcomes). **Analysis**: DIDs using year and district FE. SE clustered at district level. Covariates include individual- and district-level characteristics. Using 3 levels of district-level coverage as Tx (10%–25%, 25%–50%. >50%, omitted category: <10%). Tx defined as the district-specific proportion of all women giving birth in public facility who received JSY cash.	**Implementation issues:** No information provided. **Exposure:** JSY coverage by district ranged from <10% to >50%.	**Birth outcomes** ^7^ **Neonatal deaths/1000 live births** BL: 31 Impact w/o covariates: coverage 10%–25% vs. <10%: NS; 25%–50% vs. <10%: NS; >50% vs. <10%: –3.1 (*P* < 0.1); With covariates: NS **One-d deaths/1000 live births** BL: 15 Impact w/o covariates: coverage 10%–25% vs. <10%: NS; 25%–50% vs. <10%: NS; >50% vs. <10%: –2.0 (*P* < 0.1)With covariates: coverage 10%–25% vs. <10%: NS; 25%–50% vs. <10%: NS; >50% vs. <10%: –2.0 (*P* < 0.1) **Other outcomes** **Delivery in a health facility** BL: 39% - Impact w/o covariates: coverage >50% vs. <10%: +7.5 pp (*P* < 0.01); lower coverage: NS. With covariates: coverage >50% vs. <10%: +8.2 pp (*P* < 0.01); lower coverage: NS **Delivery in public health facility** BL: 20% - Impact w/o covariates: coverage >50% vs. <10%: +11 pp (*P* < 0.01); coverage 25%–50% vs. <10%: +1.9 pp (*P* < 0.01); lower coverage: NS. With covariates: coverage >50% vs. <10%: +10 pp (*P* < 0.01); coverage 25%–50% vs. <10%: +1.3 pp (*P* < 0.05) lower coverage: NS; **Health worker attended delivery** BL: 46% - Impact w/o covariates: coverage >50% vs. <10%: +5.6 pp (*P* < 0.01); lower coverage: NS. With covariates: coverage >50% vs. <10%: +6.3 pp (*P* < 0.01); lower coverage: NS **At least 3 ANC visits** BL: 45% - Impact: NS **Pregnancy** BL: 8.6% - Impact w/o covariates: coverage >50% vs. <10%: +0.7 pp (*P* < 0.01); lower coverage: NS. With covariates: coverage >50% vs. <10%: +0.94 pp (*P* < 0.01); lower coverage: NS	Unobserved confounding due to nonexperimental nature of study cannot be ruled out. Coverage used as the Tx variable. Coverage defined as women who gave birth in a public facility & received JSY cash/all women who delivered in a public facility. If the program encouraged noneligible women to deliver in a health facility, the treatment variable would be attenuated. Only Tx defined in % coverage categories (<10%, 10%–25%, 25%–50%, >50%) results in significant impact findings. When treatment was defined as binary variable (more than 10% coverage) or as a continuous variable (proportion between 0 and 1), no significant impact was found. Coverage may be determined by factors (other than the actual program) that also determine mortality. It cannot be ruled out that the accuracy of the mortality reporting was systematic, i.e., related to program uptake.
Nepal LBWSAT Saville et al., 2018 (38) Registration: ISRCTN75964374	**Objectives:** Improve birth outcomes (BW and child WAZ), nutrition, and sanitary practices. **Eligibility criteria and targeting:** Married and pregnant women aged 10–49 y who had not had a tubal ligation and whose husbands had not had a vasectomy; recruited women were monitored for missed menses and enrolled in the trial upon confirmed pregnancy. **Pregnancy focus:** program specifically focused on improving pregnancy outcomes.	Monthly community-based PLA women's groups on maternal and newborn health and nutrition, with and without monthly food transfers (10 kg/mo Super Cereal) or cash (∼7.5 USD/mo, equivalent to food cost) to pregnant women; home visits planned but poorly executed; control group received usual government outreach services, such as family planning, immunization, vitamin A and iron-folate distribution, health promotion, etc.	**Design**: Cluster-randomized. Study clusters were stratified by population size and accessibility; within each stratum, clusters were randomly assigned to 1 of 3 treatment arms (PLA only, PLA + food, PLA + cash) or the control arm. **Data**: Enrollment survey after confirmation of pregnancy; longitudinal follow-up at early and late pregnancy, birth, post-neonatal; cross-sectional surveys for mother & child at endpoint (all data collection 2013–2015). **Sample**: Women who delivered >16 wk after intervention started and before endpoint follow-up. ***N***: 10,936;PLA only = 2448 births;PLA + food = 2997 births;PLA + cash = 3065 births;Control = 2426 births; BW assessed in only 22% of sample. **Analysis**: ITT; linear, logistic, or ordered logistics regression with RE for cluster, adjusted for stratum and child, maternal, and household covariates.	**Implementation issues:** Food and cash distribution were delayed during monsoons due to road access problems. Cash transfers were not delivered in October 2014 because of security concerns. **Exposure:** PLA group attendance: PLA only: 2 times (49% of pregnant women attended); PLA + food: 5 times (97% of pregnant women attended); PLA + cash: 4 times (94% of pregnant women attended) Food: 4 to 5 transfersCash: 4 to 5 transfersHome visits were limited and not conducted as planned. Women in food or cash arms enrolled earlier in pregnancy and had on average 1.5 mo longer to be exposed to groups than in the PLA-only arm.	**Birth outcomes** **BW** (within 72 h): Nonbeneficiaries: 2756 g Impact: PLA only: NS; PLA + cash: NS; PLA + food: +78 g (*P* < 0.05); (difference between Tx: NS) **LBW** (<2500 g within 72 h): Nonbeneficiaries: 22.5% Impact: NS **Weight** (within 10 d): Nonbeneficiaries: 2770 g Impact: PLA only: NS; PLA + cash: +69 g (P < 0.05); PLA + food: +72 g (*P* < 0.05); (difference between Tx: NS) **Length, LAZ, head circumference** (all within 72 h), **preterm delivery:** NS **Stillbirth:** listed as outcome in methods section, but no results reported **Neonatal mortality**: dropped due to a lack of resources **Other outcomes** **Maternal weight in late pregnancy:** NS **Dietary diversity score (range: 0 to 10)** Nonbeneficiaries: 4.2 Impact: PLA only: NS, PLA + cash: +0.55 (*P* < 0.05), PLA + food: NS (difference between Tx not reported). **Eating occasions per day** Nonbeneficiaries: 3.3 Impact: PLA only: NS; PLA + cash: +0.3 (*P* < 0.01); PLA + food: NS; (difference between Tx not reported). **Delivered at health institution** Nonbeneficiaries: 34.8% Impact: PLA only: NS, PLA + cash: NS, PLA + food: OR, 1.45 (*P* < 0.05; difference between Tx not reported)	Trial disrupted due to ethnic conflict in field team leading to very low capture rates for many outcomes [e.g., only 22% of necessary BW (<72 h) assessments made], causing a drop in statistical power. Potential limited internal validity due to nonrandom attrition: women whose infants’ BW was captured were older, had more children, were more often Hindu, and had less education. Loss to follow-up appears similar across arms, but no formal analyses of differential attrition (and thus risk of selection bias) presented. Authors revised analysis plan and decided to drop certain outcomes (e.g., mortality) and to add new secondary outcomes. Lack of endpoint data for mortality prevented analysis of these outcomes. The finding that the BW effect was not sustained (as measured in the impact on WAZ at endpoint; child age 0 to 16 mo, mean: ∼9 mo) suggests that the intervention had no overall BW effect. Lack of WAZ effect may be due to endpoint data collection occurring during monsoon when levels of diarrheal disease and child underweight were high.
Mexico *Progresa/Oportunidades/Prospera* Barber & Gertler, 2008 (36) Barber & Gertler, 2010 (37) Registration: None	**Objective**: Break intergenerational transmission of poverty through investments in human capital of poor children. **Eligibility criteria and targeting:** Low-income HHs based on a proxy-means score in poor communities. **Pregnancy focus:** program not exclusively focused on pregnancy outcomes, but included components specifically aimed at improving pregnancy outcomes.	Government-implemented conditional cash transfer program. Cash transfer (∼USD 15/mo, irrespective of HH composition) conditional on participating in health education and regular check-ups; micronutrient-fortified food to pregnant and lactating women, children 4 to 23 mo; education transfer (maximum ∼$90/HH/mo and ∼$150/HH/mo, depending on age of children), conditional on regular school attendance.	**Design**: Cluster-randomized controlled trial; control clusters (communities) started receiving transfers 18 mo after Tx communities. **Data**: BL 1997; FU 2003 **Sample**: Two-stage random sample: random selection of communities (probability proportionate to number of reproductive age women). All women of reproductive age (15–49 y) who reported singleton births between 1997 and 2003. ***N*:** 840.Tx = 666 births, C = 174 births. **Analysis birth outcomes**: BW: Community FE and RE regression with individual, household, and community covariates; LBW: RE probit, RE & FE linear probability.	**Implementation issues:** An estimated 1% of HHs were denied the cash transfer due to noncompliance. **Exposure:** 97% of eligible HHs participated in program. Women in the sample participated in the program for 2.8 y before delivery, on average.	**Birth outcomes** **BW** (maternal recall) Nonbeneficiaries: 3167 g Impact: RE: +127 g (*P* < 0.05), FE: +102 g (*P* < 0.10) **LBW** (<2.500 g) Nonbeneficiaries: 10.3% Impact: RE probit: –0.323 (log odds, *P* < 0.10), RE linear probability: –4.6 pp (*P* < 0.05); FE linear probability: –4.4 pp (*P* < 0.10) **Other outcomes** **Mother sought prenatal care** (self-report) Nonbeneficiaries: 94.3% Impact: NS **Mother obtained ≥5 ANC visits** (self-report) Nonbeneficiaries: 74.2% Impact: NS **Number of ANC visits** (self-report) Nonbeneficiaries: 6.4 Impact: NS **ANC quality index** (self-report) Nonbeneficiary: 0.00 (by construction) Impact: RE: +0.36 SD (*P* < 0.01), FE: +0.41 SD (*P* < 0.01)	BW based on maternal recall; recall time longer for control than for treatment, possibly introducing differential recall bias. Data analysis did not follow original randomized design: control births included from the 1997 start of the program to the time these control areas were enrolled in the program (1.5 y later, i.e., in 1999); treatment births from 1997 to 2003 in treatment areas. Differences in outcomes may thus be due to factors other than the treatment. Last birth was observed in 2003 survey included in study. This implies that, by design, control women had higher parity than in the treatment arm. This may have affected outcomes.
Mexico *Progresa/Oportunidades/Prospera* Barham, 2011 (39) Registration: None	**Objective**: See above [Barber & Gertler, 2008 (36)]. **Eligibility criteria and targeting:** *ibidem*. **Pregnancy focus:** *ibidem*.	See above [Barber & Gertler, 2008 (36)]	**Design**: Quasi-experimental; the identification strategy exploits phasing in of municipalities over time. **Data**: Municipality-level panel with data on rural births and deaths, program participation, population, and health supply data from 1992–2001. Of the 5 phase groups (municipalities incorporated in the program from 1997 to 2001), only phases 2 and 3 were included in the analyses. **Sample:** 2391 Mexican municipalities where the program was phased in. ***N***: ∼18,000 municipality-years **Analysis**: NMR regressed on 3 treatment variables: lagged % HHs enrolled in program (main specification), lagged municipality-specific % of localities with beneficiaries, and lagged municipality having at least 1 locality with beneficiaries. Models included year and municipality FE and were weighted by number of rural HHs in municipality. Robustness checks included controlling for time-varying municipality characteristics. Impact estimated for all municipalities and by pre-program NMR and other municipality characteristics.	**Implementation issues:** No information provided. **Exposure:** No information provided.	**Birth outcomes** **Neonatal mortality rate** (death before age 30 d/1000 live births): Sample mean: 8.8 to 9.0 (depending on specification). Impact: main specification: NS; alternative specifications: -0.64 (*P* < 0.1, municipality-level variation) to –1.32 (*P* < 0.05, locality-level variation). Effect by municipality characteristics: effect limited to municipalities with above-median NMR levels: –2.50 (*P* < 0.05); effect higher in municipalities with fewer households with electricity and with larger HHs (*P* interaction < 0.01), but effect smaller in municipalities with less piped water (*P* < 0.05), more dirt floors (*P* < 0.01), and ag sector labor (*P* < 0.01). **Other outcomes** N/A	Unobserved confounding due to nonexperimental nature of study cannot be ruled out Author states that program impact is potentially underestimated because of financial intervention for beneficiaries to report deaths (missing an obligatory health appointment would lead to not receiving cash). Reporting deaths could therefore be intervention with the program. It is unlikely, however, that this would affect the estimates for NMR, as children are expected to die before the birth has been registered. Even though controlled for in 1 of the models by adding it as a covariate, it remains unclear to what extent the expansion in health-care supply in treatment areas may be driving the results.
Colombia *Familias en Acción* Attanasio et al., 2005 (41) Registration: None	**Objectives:** Decrease poverty and improve health and educational outcomes of children through preventive health care and increased school attendance. **Eligibility criteria and targeting:** *1*) Municipality <100,000 inhabitants with sufficient infrastructure; *2*) family holds Colombian citizen card; *3*) has children under 18 y; and *4*) is classified as being in the lowest level of the official socio-economic classification (SISBEN). **Pregnancy focus:** program did not include components specifically aimed at improving pregnancy outcomes.	Large-scale, government-implemented conditional cash transfer program since 2001. Nutritional subsidy (USD 15.38/mo irrespective of HH composition) for families with child 0 to 6 y, conditional on meeting basic preventive health-care intervention; school subsidy (USD 4.61 to USD 9.23 depending on age) conditional on children attending school.	**Design**: Quasi-experimental. Municipalities classified into 25 strata (based on region, population, etc.); 2 Tx municipalities randomly drawn from each stratum and each matched with a similar control municipality from the same stratum. DIDs after verifying common-trend assumption on HH labor income. **Data**: Longitudinal household data **Sample:** Municipalities with <100,000 inhabitants; HH sampling not discussed. ***N***: Not provided. **Analysis**: ITT; simple DIDs; no information on covariates or robustness checks. Analyses conducted separately for rural and urban areas.	**Implementation issues:** No information provided. **Exposure:** No information provided.	**Birth outcomes** **BW** (self-report) Rural: NS Urban: +578 g (*P* < 0.05) **Other outcomes** N/A	Not clear how matching of municipalities was done. Analytic approach lacks detailed explanation. No robustness checks shown. Unobserved confounding due to nonexperimental nature of study cannot be ruled out. Size of the impact on BW biologically implausible since women were not severely undernourished prior to intervention. BW was obtained through self-report, introducing potential recall bias. Pregnant women only eligible if they had a child under 7 years of age. Impact estimates do not include first-born children.
Uruguay *PANES* Amarante et al., 2016 (42) Registration: None	**Objectives:** Alleviate poverty among the poorest HHs in the country. **Eligibility criteria and targeting:** HHs eligible if below a “predicted income score” cutoff. The score was based on a linear combination of household socio-economic characteristics. Eligibility thresholds varied across regions. Roughly 10% of Uruguayan households enrolled. **Pregnancy focus:** program not exclusively focused on pregnancy outcomes, but included components specifically aimed at improving pregnancy outcomes.	Cash transfers (∼$56 per month, irrespective of HH composition) and electronic food card introduced midway through program (valued at 25%–50% of the cash transfer depending on HH size and composition); conditionalities (health checks for pregnant women and children) not enforced. Additional program components: public works program, training and educational activities, medical checks (including ANC visits, surgery, dental care, etc.), home improvement materials, public utilities connectivity support, assistance for small businesses, and housing for homeless families. Enrollment started in April 2005; program ended December 2007.	**Design**: Nonrandomized; fuzzy regression discontinuity design comparing “barely eligible” (just below threshold) with “barely ineligible” mothers. **Data**: All program applicants matched with 2003–2007 birth data from vital statistics and social security records (2004–2007). **Sample:** All births (2003–2007) in HHs that applied for the program (whether eligible or ineligible). ***N***: 71,811.T = 50,939 (eligible applicants); C = 20,872 (ineligible applicants). **Analysis**: localized DIDs estimator within close proximity of eligibility threshold; treatment instrumented using mother's eligibility (based on income score). All models controlled for conception month and BL month. Sensitivity analysis includes additional covariates (newborn, maternal, and HH dwelling characteristics; geographic indicators) and estimates within a narrower range of the discontinuity threshold.	**Implementation issues:** Transfer conditionalities such as attending health check-ups de facto not enforced.Transfer for food (food card) not started until mid-2006. **Exposure:** 97% of eligible households received cash transfers at some point during study period, yet only 63% during mother's pregnancy;80% of eligible households received food card at some point during study period, yet only 41% during mother's pregnancy.	**Birth outcomes** **BW**:BL: 3141 g Impact: +31 g (*P* < 0.1) **LBW** (<2500 g): BL: 10.2% Impact: –1.9 pp to –2.5 pp (*P* < 0.05)^8^ **Gestational length:** BL: 38.5 wk Impact: NS **Prematurity** (<37 wk): BL: 10.1% Impact: NS **Apgar score (range 0 to 10):** BL: 8.48 (1 min), 9.60 (5 min) Impact: +0.09 (1 min, *P* < 0.05), +0.06 (5 min, *P* < 0.05) **Other outcomes** **ANC visits:** BL: 6.5 Impact: NS **Week of first ANC visit:** BL: 17.5 wk Impact: NS **Public hospital delivery:** BL: 77% Impact: +3.1 pp (*P* < 0.05)	BW obtained from hospital records.Possible unobserved confounding due to nonexperimental nature of study. Impact estimated close to the threshold may not be generalizable to the entire eligible population. Some beneficiary households received additional program components (trainings, services, health care, etc.), i.e., program impact cannot be solely attributed to the cash transfer. Numerous robustness checks confirm results, suggesting high internal validity.

1 Outcome definitions: perinatal mortality was defined as a stillbirth after 28 weeks of pregnancy or death of a child within the first week after a live birth; stillbirth was defined as a baby born with no signs of life at or after 28 weeks of gestation. Abbreviations used: ANC, antenatal care; BL, baseline; BW, birth weight; C, control; DLHS, District Level Health Survey; DID, difference-in-difference; FE, fixed effects; FU, follow-up; HH, household; ITT, intent-to-treat; JSY, Janani Suraksha Yojana; LAZ, length-for-age z-score; N/A, not applicable; NMR, neonatal mortality rate; NS, not significant; LBW, low birth weight; LBWSAT, Low Birth Weight in South Asia Trial; OLS, ordinary least squares; PANES, Plan de Atención Nacional a la Emergencia Social; PLA, participatory learning and action; pp, percentage point; RE, random effects; SISBEN, Sistema de Identificación de Potenciales Beneficiarios para Programas; Tx, treatment; WAZ, weight-for-age z-score.

2 Program components included to improve birth outcomes are underlined.

3 Implementation issues refer to any information reported by the authors on implementation fidelity, quality of service delivery, perceptions of users and implementers, workload, and so forth.

4 Exposure refers to the utilization of services or products, frequency and duration of use, and adoption of recommended practices as reported by the authors.

5 Authors reported 95% CIs but not exact *P* values. Perinatal death was defined as a stillbirth after 28 weeks of pregnancy or death within 1 week after a live birth; neonatal death was defined as death within 1 week after a live birth.

6 Impact estimates were reported as percentages in the article, but based on an email exchange with authors, these estimates should have been pp.

7 Neonatal death was defined as death within 28 d after a live birth; 1-d death was defined as death within 24 h after a live birth.

8 Effects larger for premature children, children of unmarried mothers, and teen mothers.

### Study outcomes

The review focuses on birth outcomes as defined above. We also reported on outcomes that help understand the nutrition and other pathways of impact, to the extent that these were reported in the included studies. Our critical review (i.e., assessment of potential bias, study validity, etc.) was limited, however, to the birth outcomes. The principal summary measures were differences in means and differences in probability.

### Reporting

Impact estimates are reported with CIs and exact *P* values if reported by the authors. When authors did not report the CI, we provide the SE. If exact *P* values were not reported, we state whether they were smaller than 0.05 or 0.10.

## Results

### Search results

**FIGURE 1:**
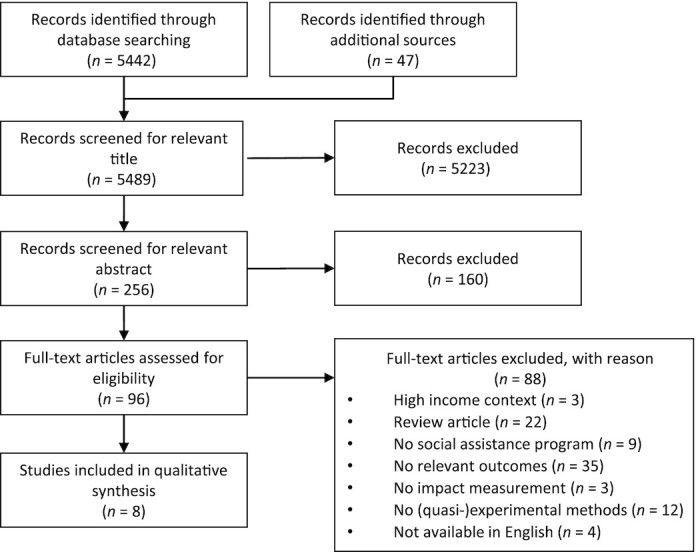
PRISMA flow diagram of studies evaluating the impact of social assistance on birth outcomes. Abbreviation: PRISMA, Preferred Reporting Items for Systematic Reviews and Meta-Analysis.

Our initial search yielded a total of 5489 articles, of which 5223 and 160 were removed after screening the title and abstract, respectively (**Figure 1**). After reviewing 96 potentially relevant full-text publications, we identified 8 studies meeting the inclusion criteria (Table 1). Since 2 of the Mexican studies were published by the same authors in different journals and only slightly differed from each other, they were considered as 1 study in this review (36, 37).

### Overview of reviewed interventions

The studies included 6 evaluations of 4 different cash transfer programs implemented in Mexico (2 studies), Colombia (1 study), India (2 studies), and Uruguay (1 study) and 1 evaluation of a community-based participatory learning and action (PLA) program in Nepal (Table 1). The programs in Mexico, Colombia, and India were conditional cash transfer programs; the program in Uruguay was de facto unconditional because the conditionalities were not enforced. Even though Uruguay is now classified as a high-income country and would therefore be excluded from this review, the program in this study was implemented in 2005–2007, when Uruguay still classified as middle-income. The remaining study evaluated the impact of community-based PLA women's groups, combined with monthly food or cash transfers, in Nepal.

Importantly, improving birth outcomes was the primary objective of only 2 of the evaluated programs (India and Nepal). Mexico's program included specific intervention components that could improve birth outcomes: the requirement to attend ANC and the provision of a micronutrient-fortified supplement for pregnant women. The programs in Colombia and Uruguay did not include any intervention component aimed specifically at improving birth outcomes. The Uruguayan program conditions, which included ANC attendance during pregnancy, were not enforced. Details on each of the interventions are provided in the **Supplemental Results**.

### Evaluation designs of the reviewed studies

All studies used experimental or quasi-experimental evaluation designs (Table 1). For the PLA study in Nepal (38), study clusters were first stratified by population size and accessibility. Within each stratum, clusters were then randomly assigned to 1 of 3 treatment arms (PLA only, PLA plus a food transfer, or PLA plus a cash transfer) or the control arm. The Nepalese trial was disrupted due to ethnic conflict in the field team. As a consequence, many outcomes were not assessed for a large percentage of study subjects. Birth weight was assessed for only 22% of the study population.

In Mexico, Barber and Gertler (36, 37) used the cluster-randomized rollout of Progresa in rural areas to estimate program impacts. Communities were randomly assigned to either receive the intervention immediately or 18 mo later. The randomized villages did not provide the necessary statistical power for Barham's (39) neonatal mortality study, so she used a quasi-experimental evaluation design with the year- and community-specific percentage of households in the Progresa program as the treatment variable.

Quasi-experimental designs were also used in the 2 studies that estimated the impacts of India's Janani Suraksha Yoijana (JSY) program (21, 40). The first study used exact matching, before-and-after comparisons, and district-level difference-in-difference analyses (21). The second JSY evaluation used only a difference-in-difference approach to estimate program impacts (40). The key difference between the 2 JSY studies is how treatment was defined in the difference-in-difference analysis: Lim et al. (21) defined it as the fraction of all births receiving JSY support in the 12 mo preceding the survey. In contrast, Powell-Jackson and colleagues (40) defined it as the proportion of women delivering in a public facility who received JSY support.

A quasi-experimental design was also used to evaluate the Familias en Acción program in Colombia (41). All municipalities in the study universe were classified in 25 strata according to location, population size, urbanicity, quality of life, and education and health infrastructure. Two municipalities receiving the program were randomly selected from each stratum, and each treatment municipality was matched with a purposively selected comparison municipality from the same stratum. Important details about study design and methods are missing in the Colombian study. The Uruguayan study (42) employed a fuzzy regression discontinuity design (a quasi-experimental approach). PANES (Plan de Atención Nacional a la Emergencia Social) eligibility depended on the household income falling below a fixed cutoff. The authors compared mothers and their newborns just below the eligibility threshold (the treatment group) to mothers and newborns just above this cutoff (the comparison group).

### Study outcomes

The most commonly reported birth outcomes were birth weight (4 studies), low birth weight (3 studies), and neonatal mortality (3 studies; Table 1). Other birth outcomes were preterm delivery (2 studies); gestational age or gestational length at birth (2 studies); weight, length, and circumference within 10 d after delivery (1 study); head circumference (1 study); stillbirths (baby born with no signs of life at or after 28 weeks of gestation, 1 study); perinatal mortality (stillbirth or death of the child within the first week after a live birth, 1 study); 1-d mortality (death within 24 h after birth, 1 study); and the Apgar score (1 study).

In terms of nutrition pathways, only 1 study assessed maternal dietary intake during pregnancy; 2 other studies assessed this outcome indirectly. Some of the studies also looked at pathways related to health service utilization outcomes, such as the number of ANC visits (4 studies) and delivery at a health facility (4 studies). No outcomes related to nutrition and health pathways were assessed in the studies.

### Impacts on birth weight and low birth weight

The 4 studies assessing birth weight found a consistent, positive impact on this outcome. In Nepal, the effect on birth weight (measured within 72 h after birth) was limited to the PLA-plus-food arm, in which a 78 g impact (95% CI: 15.6–140.5; *P* = 0.0143) was found; the point estimate of the impact in the PLA-plus-cash arm (50 g) was not significantly different from 0, likely because of the considerable loss in statistical power following severe attrition due to the problems in the field team (38). No birth-weight effect was found in the PLA-alone arm. Given the large loss to follow-up (78%), the authors also estimated the impact on newborn weight by using all weights measured within 10 d after birth, which were available for 27% of eligible newborns. Significant program effects on this outcome were found in the PLA-plus-cash arm (69 g; 95% CI: 3.2–134.4; *P* = 0.0397) and the PLA-plus-food arm (72 g; 95% CI: 7.5–137.2; *P* = 0.0288), but not in the PLA-only arm. The intervention had no impact on the prevalence of low birth weight.

Barber and Gertler (36, 37) found that Progresa had a positive effect on birth weight, ranging from 102 g (SE = 58.3; *P* < 0.10) to 127 g (95% CI: 21.3–233.1; *P* = 0.02); the program reduced the prevalence of low birth weight by an estimated 4.4 (SE = 0.025; *P* < 0.1), to 4.6 percentage points (SE = 0.024; *P* = 0.05). With a low-birth-weight prevalence in the control group of 10.3%, this suggests that the program almost halved the prevalence of low birth weight. In Colombia, the impact of the Familias en Acción program on birth weight was limited to urban areas (41), but we deem the estimated impact of 578 g (SE = 0.143; *P* < 0.05) to be biologically implausible since women in the study were not severely undernourished prior to the intervention. PANES in Uruguay was found to have a positive effect on birth weight of 31 g (SE = 18.4; *P* < 0.1) (42). It reduced the prevalence of low birth weight by 1.9 percentage points (SE = 0.007; *P* < 0.01), to 2.5 percentage points (SE = 0.011; *P* < 0.05), equivalent to a 20% reduction of the preprogram prevalence. Additional analyses showed that the program's impact on birth weight was larger for premature children, children of unmarried mothers, and teen mothers. Across the 4 studies, the overall certainty of evidence on birth weight and low birth weight was rated as very low (**Table 2**; Supplemental Results).

**TABLE 2:** Assessment of certainty of evidence using the GRADE approach of studies evaluating the impact of social assistance on birth outcomes^1^

Outcome (*n* of each study type)	Limitations	Consistency	Directness	Precision	Publication bias	Overall certainty of evidence^2^
Birth weight(2 RCTs, 2 quasi-experimental studies)	Very serious limitations Severe loss to follow-up (38) Differences in recall periods between treatment and control may have biased findings (36, 37) Confounding due to health-care expansion (36, 37) Missing details on design and methods (41)	Serious inconsistency Impact estimates ranged from 31 to 578 g Largest effect is biologically implausible (41)	No serious indirectness	No serious imprecision	Likely publication bias Primary outcome (registered) in only 1 study (37) Decision to publish the results of secondary impact analyses possibly influenced by significance of the estimates.	Very low
Neonatal mortality(3 quasi-experimental studies)	Very serious limitations Confounding due to health-care expansion (39) Challenge of defining treatment, possible reverse causality, accuracy of the mortality measure possibly associated with program uptake (21, 40)	Serious inconsistency Impact estimates ranged from not significant to 15% Statistical power calculations not provided	No serious indirectness	Serious imprecision	Likely publication bias Not a primary or registered outcome in any of the studies Decision to publish the results of secondary impact analyses possibly influenced by significance of the estimates.	Very low

1 Additional details are provided in the text. Abbreviations: GRADE, Grading of Recommendations, Assessment, Development and Evaluations; RCT, randomized controlled trial.

2 GRADE Working Group grades of evidence are as follows. High quality indicates that we are very confident that the true effect lies close to that of the estimate of the effect. Moderate quality indicates that we are moderately confident in the effect estimate: the true effect is likely to be close to the estimate of the effect, but there is a possibility that it is substantially different. Low quality indicates that our confidence in the effect estimate is limited: the true effect may be substantially different from the estimate of the effect. Very low quality indicates that we have very little confidence in the effect estimate: the true effect is likely to be substantially different from the estimate of effect.

### Impact on mortality

Evidence on neonatal mortality was found in India and Mexico (21, 39, 40). Neonatal mortality was dropped as an outcome from the Nepalese study due to the challenges faced while implementing the study. The 2 studies estimating the impact of the JSY program in India found evidence of an impact on perinatal and neonatal mortality: Lim and colleagues (21) found reductions in perinatal mortality of 3.7 (95% CI: –5.2 to –2.2; *P* < 0.05) to 4.1 (95% CI: –5.7 to –2.5; *P* < 0.05) deaths/1000 pregnancies in 2 of the 3 model specifications (the difference-in-difference model did not demonstrate a significant program impact), equivalent to a 9% to 10% reduction. The estimated reduction in neonatal mortality (significant in the same 2 model specifications) was 2.3 (95% CI: –3.7 to –0.9; *P* < 0.05) to 2.4 (95% CI: –4.1 to –0.7; *P* < 0.05) deaths/1000 live births, equivalent to a 7% reduction. Interestingly, the largest mortality reductions were found in the non-high-focus states, which were defined as having low in-facility birth coverage (Table 1). In contrast, Powell-Jackson et al. (40) found that the impact of JSY was limited to districts with coverage above 50%. In these districts, neonatal mortality was 3.1 deaths/1000 live births lower (SE = 0.0016; *P* < 0.1), a 10% reduction compared to districts with a coverage level below 10%. When covariates were added to the model, however, the point estimates were no longer significant. The effect on 1-d mortality was a decrease of 2 deaths per 1000 live births (SE = 0.0012; *P* < 0.1), equivalent to a 13% reduction. This estimate did not change when covariates were added to the model. No impact was found at coverage levels lower than 50%.

In rural Mexico, the estimated effect of Progresa on neonatal mortality ranged from –0.64 (SE = 0.39; *P* < 0.1) to –1.32 (*P* < 0.1) deaths/1000 live births, depending on the model specification (39). This indicates that the program may have reduced neonatal mortality by about 1 neonatal death per 1000 live births, representing a 10% reduction in the neonatal mortality rate (given a rate of about 9 deaths per 1000 live births in the total sample). The effect was limited, however, to municipalities where the preprogram neonatal mortality rate was above the sample median. In these municipalities, the program reduced mortality by an estimated 2.5 neonatal deaths/1000 live births (SE = 0.88; *P* < 0.05), equivalent to a 19% reduction. The analysis of effect modifications by municipality characteristics resulted in inconsistent findings. The program impact was higher in municipalities with fewer households with electricity or in those with larger households (*P* for interaction < 0.01). The impact on mortality was smaller, however, in municipalities where fewer households had access to piped water (P for interaction < 0.05), in those with more households with dirt floors (*P* < 0.01), and in those with a larger proportion of the population working in the agricultural sector (*P* < 0.01). The certainty of the evidence for neonatal mortality was graded as very low (Table 2; Supplemental Results).

### Impact on other birth outcomes

No impact was found on gestational length or prematurity in the Nepalese and Uruguayan studies where these outcomes were measured. No impact was found in Nepal on length or head circumference measured within 10 d after birth. Even though listed in the methods section of this study, no statistical analysis on stillbirths was reported. In Uruguay, the impacts on the 1- and 5-min Apgar scores were 0.09 (SE = 0.037; *P* < 0.05) and 0.06 (SE = 0.027; *P* < 0.05), respectively.

### Impact on nutrition outcomes along the impact pathways

In Nepal, the impact on diet-related outcomes was limited to the PLA-plus-cash arm: women's dietary diversity score in this arm was 0.55 food groups higher compared to the control arm (95% CI: 0.12–0.99; *P* = 0.013). The effect on the number of eating occasions per day in this arm was +0.3 (95% CI: 0.1–0.5; *P* = 0.007). Barber and Gertler (36, 37) used the time spent as a Progresa beneficiary as a proxy for the cumulative effect of the program's fortified food and of improvements in the household diet as a consequence of behavior change communication and the cash transfer. The lack of a statistically significant association between birth weight and length of program exposure led the authors to conclude that the birth-weight effect did not operate through improvements in nutritional status. Given the absence of an effect on care seeking during pregnancy in the Uruguayan program, the authors concluded that the effect on birth weight must be driven by improvements in maternal nutrition during pregnancy due to the cash transfers.

### Impact on health outcomes along the impact pathways

The 2 evaluations of India's JSY program found mixed results on ANC care seeking: Lim et al. (21) found a statistically significant effect on the proportion of women with a least 3 ANC visits [+11 percentage points (pp) in each of the 3 model specifications], but no effect was found by Powell-Jackson et al. (40). In line with the program design, positive effects were found on delivery in a health facility and births attended by a skilled birth attendant. Lim et al. (21) found large effects on the proportion of women delivering in a health facility (+44 to 49 pp across the 3 model specifications) and the proportion of births attended by a skilled birth attendant (+36 to 39 pp idem). The second JSY evaluation (40) estimated that in districts with at least 50% coverage, delivery in a health facility increased by 7.5 pp [SE = 0.0093; *P* < 0.01; 8.2 pp when controlling for covariates (SE = 0.0084; *P* < 0.01)], delivery in a public health facility increased by 11 pp [SE = 0.0086; *P* < 0.01; 10.0 pp idem (SE = 0.0084; *P* < 0.01)], and health worker–attended deliveries increased by 5.6 pp [SE = 0.0090; *P* < 0.01; 6.3 pp idem (SE = 0.0081; *P* < 0.01)].

In Nepal, where a significant effect on birth weight measured within 10 d was found in the PLA-plus-cash and PLA-plus-food arms, women in the PLA-plus-food arm were significantly more likely compared to women in the control arm to deliver at a health institution (OR, 1.45; 95% CI: 1.03–2.06; *P* = 0.0344). No effect on place of delivery was found in the PLA or PLA-plus-cash arms.

Barber and Gertler (37) found no impact of Mexico's Progresa program on ANC seeking, which was already high (6.4 visits per pregnancy) in the absence of the program. The authors reported that the program had a positive effect on the quality of the care received. The assessment of quality, however, was based on mothers’ recall of the different procedures received. The authors concluded that the impact on birth weight was due to the higher quality of the prenatal care received, which was in turn a consequence of empowering women to negotiate better care from health-care providers. Although the Progresa program was shown to increase women's empowerment (43, 44), the program's impacts on quality of care or of women's negotiation skills with health-care providers were not directly measured. The Uruguayan program did not have a positive effect on ANC seeking but increased public hospital delivery by 3.1 pp (SE = 0.015; *P* < 0.05).

## Discussion

We reviewed the literature on the impacts of social assistance programs on birth outcomes in low- and middle-income countries. The 4 studies that assessed birth weight found positive impacts on this outcome (36–38, 41, 42), which ranged from 31 g to 578 g. Of the 3 studies that reported on neonatal mortality, 2 documented significant effects on this outcome, with estimated reductions ranging from 0.6 to 3.1 deaths per 1000 live births (21, 39, 40). Except for significant effects on 1- and 5-min Apgar scores in 1 study (42), none of the other birth outcomes reported in the reviewed studies were found to benefit from the social assistance programs.

In spite of the relatively low certainty of the evidence, the size of the estimated impacts on birth weight and neonatal mortality is clinically relevant. A meta-analysis of energy and protein supplementation interventions during pregnancy found an increase in birth weight of 41 g (6). Multiple-micronutrient supplementation had a similar effect on this outcome (38 to 40 g) (45). The birth-weight effects of nutrition education to increase energy and protein intakes appear to be limited to undernourished women: 2 small trials showed an effect of 490 g (6). Known effective interventions aimed at lowering neonatal mortality have effect sizes that vary from 0.36 to 1.55 deaths/1000 live births (20).

Evidence on the nutritional pathways of impact in the reviewed studies was limited. Only 1 of the studies, the evaluation of the Nepalese intervention (38), assessed maternal diet–related outcomes. The impact on women's dietary diversity during pregnancy and on the number of daily eating occasions was limited to the PLA-plus-cash arm, even though the intervention's effect on birth weight was found in both the PLA-plus-cash and the PLA-plus-food arms. Barber and Gertler (36, 37) used the time spent as a Progresa beneficiary as a proxy for the cumulative effect the program could have had on beneficiary women's nutritional status through the consumption of the fortified food, exposure to behavior change communications, and improvements in the household diet as a consequence of the cash transfer. They regressed birth weight on program months and concluded, based on the statistical insignificance of the regression coefficient, that the program impact did not operate through improvements in maternal nutritional status. It is unclear, however, whether the study was powered to detect an association between time in the program and birth weight. In addition, it is possible that the association between program months and birth weight is not linear; misspecification of the functional form may thus explain the lack of an association, too.

Evidence on health pathways was mixed. JSY in India increased the proportion of women with at least 3 ANC visits even though the program did not promote the use of ANC (21, 40). Mexico's conditional cash transfer program required pregnant women to attend at least 5 ANC visits, but no effect on this outcome was found because of the already high number of visits (6.4) in the absence of the program (37). The ANC requirement in Uruguay was never enforced, and no impact was found on this outcome (42). As would be expected given the program conditionality, an increase in delivering in a health facility was observed in the JSY program in India (21, 40). Barber and Gertler (36, 37) concluded that the impact on birth weight in Mexico was due to the higher quality of the prenatal care received, which in turn was due to women being more empowered to demand better care from health-care providers. This conclusion, however, was not supported by data. Even though the program had an impact on women's empowerment (43, 44), there is no evidence that women were more empowered to “demand” better health services. In addition, the measure of health services quality was solely based on women's recall. Finally, which biological mechanism could have resulted in an impact on birth weight of over 100 g as a result of better-quality ANC visits is unclear. A positive impact on facility delivery was found in 1 of the arms in Nepal, where facility delivery was promoted through the PLA, and in the Uruguayan program, which did not include any program activity targeting this outcome (38, 42).

What are the implications of our findings for policy and research? Even though the quality of the reviewed studies varied, the evidence suggests that social assistance programs can improve birth outcomes. This conclusion is supported by other evidence on their effectiveness. Cash transfer programs have been demonstrated to be an effective policy tool to reduce poverty, improve household food security, and increase spending on nutrient-rich foods; increase health care–seeking behavior, especially when health visits are a program requirement; have been shown to improve psychosocial health and to reduce domestic violence by a male partner; and may increase women's social capital and decision-making power (11, 12, 15, 46, 47). Social assistance programs thus hold tremendous promise to improve birth outcomes through improvements in income, food security, and household consumption of nutritious foods, and more directly through improvements in pregnant women's nutritional and physical and mental health statuses and increased use of ANC services and skilled birth attendants.

Following the WHO recommendation to expand research on the effectiveness of cash transfers and related approaches for improving birth outcomes, and given that existing evidence is promising but has very low certainty, carefully designed impact evaluations are needed to quantify the effects of social assistance programs on birth outcomes. Quasi-experimental studies are more feasible than randomized trials for mortality outcomes, since the detection of a meaningful mortality effect requires a very large sample size. This makes sufficiently powered randomized mortality trials prohibitively expensive to conduct, especially since social assistance interventions are difficult to randomize at the individual level; cluster randomization further increases sample size requirements. However, the use of randomized designs to evaluate the impacts of social assistance programs on other birth outcomes, such as birth weight, preterm delivery, and being small for gestational age, is achievable and would strengthen the evidence base. Even though the authors of the quasi-experimental designs included in this review used a variety of methods to reduce selection bias, the possibility of this bias affecting the results cannot be excluded (see last column in Table 1). The biologically implausible birth-weight impact of nearly 600 g in the Colombian study may be a consequence of this problem (41). Studies should attempt to measure birth outcomes directly rather than through maternal recall. Impact evaluations also need to be adequately powered to detect meaningful improvements in birth outcomes. Only 1 of the studies (the evaluation of the Nepal program) conducted ex ante power calculations. The absence of information on the minimum effects the studies were powered to detect makes it impossible to assess whether impact estimates that are not significantly different from 0 reflect a true absence of impact or are simply due to the study not being sufficiently powered.

Evaluation studies should also elucidate the underlying mechanisms of impact to confirm the plausibility of findings and to adapt programs and increase their effectiveness. Since micro- and macronutrient deficiencies are important determinants of poor birth outcomes, assessing the impact of programs on dietary adequacy during pregnancy is important. Evaluations should also measure the impacts on ANC seeking, the quality of care women receive, and women's empowerment in nutrition and health domains.

A number of other considerations would allow evidence to better inform policies and programs. Evaluation studies need to be conducted on a variety of programs in different settings to understand how intervention characteristics and contexts affect the sizes of the impacts on birth outcomes. Studies could be designed to better understand the differential effects of including pregnancy-specific behavior change communication and counseling and of using social protection programs to distribute special micronutrient-fortified foods and micronutrient supplements. Impact studies should also assess potential unintended negative consequences on outcomes, such as a decline in service quality of ANC, reduced pregnancy spacing, and excess pregnancy weight gain and subsequent weight retention during the postpartum period, as recently shown in Guatemala (48). Finally, we recommend that impact evaluation studies also measure the financial and economic program costs. This will allow donors and policy-makers to compare the costs and benefits of social assistance programs to the costs and benefits of other solutions to improve birth outcomes.

## Supplementary Material

nxab292_Supplemental_File

## Notes

Supported by the Bill & Melinda Gates Foundation (grant no. OPP1172421) and the CGIAR Research Program on Agriculture for Nutrition and Health (A4NH), led by the International Food Policy Research Institute.

Author disclosures: The authors report no conflicts of interest.

The funders played no role in the study design and implementation and in the analysis and interpretation of the data.

Supplemental Results and Supplemental Methods are available from the “Supplementary data” link in the online posting of the article and from the same link in the online table of contents at https://academic.oup.com/jn/.

Abbreviations used: ANC, antenatal care; GRADE, Grading of Recommendations, Assessment, Development and Evaluations; JSY, Janani Suraksha Yoijana; PANES, Plan de Atención Nacional a la Emergencia Social; PLA, participatory learning and action; pp, percentage points; PRISMA, Preferred Reporting Items for Systematic Reviews and Meta-Analyses.
