# Supplementary material for: Social Assistance Programs and Birth Outcomes: A Systematic Review and Assessment of Nutrition and Health Pathways
Source: J Nutr. 2021 Sep 29;151(12):3841–55. doi: 10.1093/jn/nxab292 (PMC8643580; doi:10.1093/jn/nxab292)
Supplement: nxab292_Supplemental_File [file nxab292_supplemental_file.docx]

**Supplemental Methods**

**Data sources and search strategies** – The search strategy combined a group of “exposure” terms (such as safety net, social transfer, food transfer, cash transfer, voucher) with “outcome” terms (such as gestational weight gain, birthweight, low birthweight, small-for-gestational-age, prematurity), “subject” terms (such as mother, newborn, infant), and “context” terms (such as low-income country). Our primary searches were conducted in three databases, PubMed, EconLit, and Google Scholar. The databases were last searched on March 20, 2019. We used the following Boolean search phrase: (safety net OR safety-net OR social transfer OR food transfer OR cash transfer OR voucher OR take-home rations OR modalities OR transfers OR social assistance OR social protection OR incentive payments OR conditional cash transfer OR care-seeking OR care-seeking OR behavior change OR behavior change) AND (gestational weight gain OR birthweight OR birth weight OR birth-weight OR low birthweight OR low birth-weight OR low birth weight OR very low birth weight OR small for gestational age OR small-for-gestational-age OR prematurity OR birth outcomes OR gestational length OR nutrition OR nutritional status OR postpartum OR prenatal OR perinatal) AND (mother OR newborn OR infant OR neonatal OR pregnant women OR maternal OR pregnancy AND (low-income country OR low socioeconomic status OR lower socioeconomic status OR low income). In addition to the articles found through the database searches, we included studies that we identified while reviewing the reference lists of included studies and existing literature reviews.

**Supplemental Results**

**Details on the interventions evaluated by the included papers**

Two studies evaluated the impacts of *Janani Suraksha Yojana* (*JSY*, or “Safe Motherhood Scheme” in English) in India (5,6). With the objective of reducing maternal and neonatal mortality, this national cash transfer program provided a one-time cash transfer to women of low socioeconomic status conditional on their giving birth in government or accredited private health facilities. Women were also offered ANC services during pregnancy. To provide greater financial incentives to disadvantaged women in areas with higher mortality rates, the transfer amount varied by state and rural vs. urban areas, ranging from around USD 13 to 31 (2005 USD; no information provided on the share of household expenditure). In addition, as incentive to frontline workers to encourage women to deliver in a health facility, these workers received approximately USD 4 to 13 for each birth they attended.

In Nepal, Saville *et al.* conducted a study on the impact of community-based participatory learning and action (PLA) women’s groups (7). The program was targeted to pregnant women in rural areas and aimed at improving birthweight and other neonatal outcomes through increased intake of nutritious food during pregnancy. The PLA groups focused on maternal and newborn health and nutrition and encouraged women to attend antenatal care and seek institutional delivery. In addition to participating in the PLA groups, pregnant women in two of the intervention arms received either a monthly food ration (10 kg of super cereal, which is a micronutrient-fortified wheat-soy blend) or an unconditional monthly cash transfer (USD 7.5, 2014 USD) equivalent to two days of wage labor. Home visits were planned as well, but implementation of this component was limited due to work overload of field mobilizers.

Barber & Gertler and Barham estimated the impact of *Progresa* (later known as *Oportunidades* and then rebranded as *Prospera*; for simplicity, we use *Progresa* throughout) on birth outcomes in Mexico (8–10). *Progresa* was Mexico’s flagship poverty reduction program (11) reaching more than 5 million low-income (mostly rural) households. The program provided cash transfers, conditional on the household complying with health and education requirements, and micronutrient-fortified foods targeted to specific household members. Specifically, households received a monthly base transfer fixed at around USD 11 (1997 USD) regardless of household size that was conditional on family members participating in preventive health care activities such as immunizations, well-baby care, and growth monitoring of children, pre- and postnatal care and education for women, health check-ups for other family members, and health and nutrition education. The monthly education transfer was conditional on children attending 85% of school days and varied by school grade and gender. The maximum total monthly transfer ranged from approximately USD 68 to 87 (1997 USD) for families with primary and high school children, respectively. On average, the transfers were equivalent to approximately 20% of total household consumption (12). Two different micronutrient-fortified foods were developed. The first was targeted to pregnant and lactating women and the second to all children 6 to 23 mo of age and to low-weight children 24 to 47 mo of age.

*Familias en Acción*, a conditional cash transfer program in Colombia similar to Mexico’s *Progresa program,* was evaluated by Attanasio *et al.* (13). The program sought to increase school attendance and improve the health and nutrition of children by providing cash transfers to households in the poorest 20% of the Colombian population conditional on health and education requirements. Monthly transfers of approximately USD 15 (or around 24% of household expenditure (14); 2002 USD) were provided to mothers with a child under 7 y of age, i.e. no cash was provided to pregnant women who did not have children in this age group. The transfers were conditional on mothers participating in health and nutrition education sessions and children attending preventive health check-ups. Similar to *Progresa,* additional cash was given to families with school-aged children (6 to 17 y old) conditional on their attending at least 80% of classes. These education transfers ranged from roughly USD 5 to 9 (2002 USD), depending on the children’s age. Cash was transferred directly to women.

Amarante *et al.* studied the impact of the Uruguayan social assistance program *Plan de Atención Nacional a la Emergencia Social* (*PANES*) (15). Aimed at alleviating poverty, this program was targeted to the poorest 10% of the population and consisted of monthly cash transfers, electronic food cards, a voluntary public works employment program as well as a wide range of other components that were received by only a fraction of all beneficiaries. The program was intended to be conditional on participation in health checks for pregnant women and children, as well as on school attendance. These conditionalities, however, were not enforced which made the transfers de facto unconditional. Households received either the monthly cash transfers of USD 56 (2005 USD; approximately 25% of pre-program mean household expenditure) or participated in the voluntary public works employment program, which paid a monthly salary of USD 107 (2005 USD). The food cards were introduced halfway through the program and ranged from one-fourth to one-half of the value of the cash transfer, depending on household size and demographic structure. Overall, only around 13% of beneficiary households received at least one of the additional program components, such as training and educational activities, medical checks (including ANC visits, surgery, dental care, etc.), home improvement materials, public utilities connectivity support, assistance for small businesses, and housing for homeless families.

**Assessment of certainty using the GRADE approach for birthweight (Table 2)**

- **Study design and risk of bias:** Only two (Nepal, Mexico) of the four studies were randomized trials (7–9) one of which (Nepal) suffered from severe loss-to-follow up (7). Attritted mothers were significantly younger, had fewer children, were less likely to be Hindu than Muslim, and were more likely to have primary or secondary education, but it is not clear whether these characteristics differed systematically across arms. Several factors may affect the internal validity of the Mexico trial as well (8,9): The 2003 survey used in the study asked women about the most recent pregnancy. Births, however, were drawn from a much shorter period in the control arm (1997 to 1999 or 1.5 y) than in the treatment (1997 to 2003 or 6 y) areas, opening the possibility that time-related factors drive the findings. The different recall periods for self-reported birthweight in control and treatment areas may have introduced bias. Because data on the most recent birth were used, control women had higher parity than women in the treatment arm, which may have affected outcomes as well. The other two studies (Columbia, Uruguay) used quasi-experimental designs, which increases the possibility of confounding (13,15). Important details about study design and methods were missing in the Colombia study (13). Moreover, only the Nepal study assessed birthweight directly (7); the other studies used maternal recall (Mexico, Columbia: 37,38,42) or hospital records (Uruguay: 44). This is less precise, but it is not clear if it could have introduced bias.
- **Consistency:** The findings were highly inconsistent as impact estimates ranged from 31 g to 578 g (the latter from Colombia (13) being implausible). The second largest estimates (102 g to 127 g) come from the *Progresa* evaluation in Mexico (8,9) and were, based on the authors’ analyses, attributable to the higher quality of care beneficiary women demanded (a hypothesized consequence of the program empowering women to negotiate better care from healthcare providers) and not to improvements in dietary intake. Beneficiary women were more likely to report having a urine sample or blood pressure taken. It is not clear, however, which biological mechanisms would underlie this effect size of over 100 g.
- **Directness:** The study populations, interventions, and study outcomes were identical to those of interest.
- **Precision:** We did not calculate a or a corresponding confidence interval because of the small number of studies and the heterogeneity in evaluation designs used. The total number of participants in the included studies was large, however, which helps increase the precision of the estimates.
- **Publication bias**: Birthweight was the primary outcome in the Nepal study, which was the only one with registered trial outcomes (7). The decision for authors of the other studies to publish the results of secondary impact analyses on birthweight may have been influenced by the significance of the estimates.

**Assessment of certainty using the GRADE approach for neonatal mortality**

- **Study design and risk of bias:** All three studies used quasi-experimental designs. Key limitations of the *JSY* evaluations were the challenge of defining treatment, the possibility of reverse causality, and the possibility that the accuracy of the mortality measure was associated with program uptake (5,6). A possible confounding factor in Mexico was the expansion of the healthcare supply (10).
- **Consistency:** The findings were highly inconsistent as impact estimates in absolute terms ranged from statistically insignificant to 4.1 deaths per 1,000 livebirths (not considering subgroup analyses), and in relative terms from statistically insignificant to a significant reduction in the mortality rate of 15% (*idem*). None of the studies provides information on statistical power, making it impossible to assess whether the statistically insignificant results reflect “true” zero effects or a lack of power. In Mexico, the largest reduction in neonatal mortality was found in areas with high mortality rates whereas in India the largest mortality effect was found in better-off states (5,10).
- **Directness:** The study populations, interventions, and study outcomes were identical to those of interest.
- **Precision:** Because of the small number of studies and the heterogeneity of their methods, we did not calculate a summary effect size and a corresponding confidence interval. The total number of participants in the included studies, however, was large.
- **Publication bias:** None of the studies on neonatal mortality registered outcomes in a trial registry, so the decision to publish findings on this outcome were likely influenced by the significance of the estimates, although some of the studies did report insignificant results.

**References**

1. Ota E, Hori H, Mori R, Tobe-Gai R, Farrar D. Antenatal dietary education and supplementation to increase energy and protein intake. Ota E, editor. Cochrane database Syst Rev [Internet]. Chichester, UK: John Wiley & Sons, Ltd; 2015;CD000032. Available from: http://doi.wiley.com/10.1002/14651858.CD000032.pub3

2. Heidkamp R, Clermont A, Phillips E. Modeling the Impact of Nutrition Interventions on Birth Outcomes in the Lives Saved Tool (LiST). J Nutr [Internet]. 2017;147:2188S-2193S. Available from: http://www.ncbi.nlm.nih.gov/pubmed/28904112

3. Keats EC, Haider BA, Tam E, Bhutta ZA. Multiple-micronutrient supplementation for women during pregnancy. Cochrane database Syst Rev [Internet]. 2019;3:CD004905. Available from: http://www.ncbi.nlm.nih.gov/pubmed/30873598

4. Bourassa MW, Osendarp SJM, Adu‐Afarwuah S, Ahmed S, Ajello C, Bergeron G, Black R, Christian P, Cousens S, Pee S, et al. Review of the evidence regarding the use of antenatal multiple micronutrient supplementation in low‐ and middle‐income countries. Ann N Y Acad Sci [Internet]. 2019;1444:6–21. Available from: https://onlinelibrary.wiley.com/doi/abs/10.1111/nyas.14121

5. Lim SS, Dandona L, Hoisington JA, James SL, Hogan MC, Gakidou E. India’s Janani Suraksha Yojana, a conditional cash transfer programme to increase births in health facilities: an impact evaluation. Lancet. 2010;375:2009–23.

6. Powell-Jackson T, Mazumdar S, Mills A. Financial incentives in health: New evidence from India’s Janani Suraksha Yojana. J Health Econ. North-Holland; 2015;43:154–69.

7. Saville NM, Shrestha BP, Style S, Harris-Fry H, Beard BJ, Sen A, Jha S, Rai A, Paudel V, Sah R, et al. Impact on birth weight and child growth of Participatory Learning and Action women’s groups with and without transfers of food or cash during pregnancy: Findings of the low birth weight South Asia cluster-randomised controlled trial (LBWSAT) in Nepal. PLoS One [Internet]. 2018;13:e0194064. Available from: http://www.ncbi.nlm.nih.gov/pubmed/29742136

8. Barber SL, Gertler PJ. The impact of Mexico’s conditional cash transfer programme, Oportunidades, on birthweight. Trop Med Int Health [Internet]. 2008;13:1405–14. Available from: http://doi.wiley.com/10.1111/j.1365-3156.2008.02157.x

9. Barber SL, Gertler PJ. Empowering women: how Mexico’s conditional cash transfer programme raised prenatal care quality and birth weight. J Dev Eff. 2010;2:51–73.

10. Barham T. A healthier start: The effect of conditional cash transfers on neonatal and infant mortality in rural Mexico. J Dev Econ. North-Holland; 2011;94:74–85.

11. Levy S. Progress against poverty: sustaining Mexico’s Progresa-Oportunidades program [Internet]. Washington, D.C.: Brookings Institution Press; 2006. xi, 166 p. Available from: http://www.loc.gov/catdir/toc/ecip0620/2006028230.html

12. Hoddinott J, Skoufias E. The Impact of PROGRESA on Food Consumption. Econ Dev Cult Change. International Food Policy Research Institute; World Bank; 2004;53:37–61.

13. Attanasio OP, Gomez LC, Heredia P, Vera-Hernandez M. The short-term impact of a conditional cash subsidy on child health and nutrition in Colombia. London: Centre for the Evaluation of Development Policies, Institute for Fiscal Studies; 2005;

14. Leroy JL, Ruel MT, Verhofstadt E. The impact of conditional cash transfer programmes on child nutrition: a review of evidence using a programme theory framework. J Dev Eff. 2009;1:103–29.

15. Amarante V, Manacorda M, Miguel E, Vigorito A. Do Cash Transfers Improve Birth Outcomes? Evidence from Matched Vital Statistics, and Program and Social Security Data. Am Econ J Econ Policy [Internet]. 2016;8:1–43. Available from: http://pubs.aeaweb.org/doi/10.1257/pol.20140344
